# *Legionella* effector AnkX interacts with host nuclear protein PLEKHN1

**DOI:** 10.1186/s12866-017-1147-7

**Published:** 2018-01-05

**Authors:** Xiaobo Yu, Rebecca R. Noll, Barbara P. Romero Dueñas, Samual C. Allgood, Kristi Barker, Jeffrey L. Caplan, Matthias P. Machner, Joshua LaBaer, Ji Qiu, M. Ramona Neunuebel

**Affiliations:** 1 State Key Laboratory of Proteomics, Beijing Proteome Research Center, National Center for Protein Sciences-Beijing (PHOENIX Center), Beijing Institute of Radiation Medicine, Beijing, 102206 China; 2 Department of Biological Sciences, University of Delaware, 105 The Green, Newark, DE 19716 USA; 3 Virginia G. Piper Center for Personalized Diagnostics, Biodesign Institute, Arizona State University, Tempe, AZ 85287 USA; 4 Cell Biology and Neurobiology Branch, Eunice Kennedy Shriver National Institute of Child Health and Human Development, National Institutes of Health, Bethesda, MD 20892 USA; 5 Delaware Biotechnology Institute, University of Delaware, Newark, 19716 DE USA

**Keywords:** Nucleic acid programmable protein array, AnkX, PLEKHN1

## Abstract

**Background:**

The intracellular bacterial pathogen *Legionella pneumophila* proliferates in human alveolar macrophages, resulting in a severe pneumonia termed Legionnaires’ disease. Throughout the course of infection, *L. pneumophila* remains enclosed in a specialized membrane compartment that evades fusion with lysosomes. The pathogen delivers over 300 effector proteins into the host cell, altering host pathways in a manner that sets the stage for efficient pathogen replication. The *L. pneumophila* effector protein AnkX targets host Rab GTPases and functions in preventing fusion of the *Legionella*-containing vacuole with lysosomes. However, the current understanding of AnkX’s interaction with host proteins and the means through which it exerts its cellular function is limited.

**Results:**

Here, we investigated the protein interaction network of AnkX by using the nucleic acid programmable protein array (NAPPA), a high-density platform comprising 10,000 unique human ORFs. This approach facilitated the discovery of PLEKHN1 as a novel interaction partner of AnkX. We confirmed this interaction through multiple independent in vitro pull-down, co-immunoprecipitation, and cell-based assays. Structured illumination microscopy revealed that endogenous PLEKHN1 is found in the nucleus and on vesicular compartments, whereas ectopically produced AnkX co-localized with lipid rafts at the plasma membrane. In mammalian cells, HaloTag-AnkX co-localized with endogenous PLEKHN1 on vesicular compartments. A central fragment of AnkX (amino acids 491–809), containing eight ankyrin repeats, extensively co-localized with endogenous PLEKHN1, indicating that this region may harbor a new function. Further, we found that PLEKHN1 associated with multiple proteins involved in the inflammatory response.

**Conclusions:**

Altogether, our study provides evidence that in addition to Rab GTPases, the *L. pneumophila* effector AnkX targets nuclear host proteins and suggests that AnkX may have novel functions related to manipulating the inflammatory response.

**Electronic supplementary material:**

The online version of this article (10.1186/s12866-017-1147-7) contains supplementary material, which is available to authorized users.

## Background

Intracellular bacterial pathogens have devised a multitude of strategies to successfully proliferate within eukaryotic cells. Paradoxically, bacterial pathogens have the ability to thrive in cells of the innate immune system (macrophages, neutrophils, and dendritic cells), which are normally destined to ingest and digest microbes [1]. Within these phagocytic cells, pathogens often form protective niches, exploiting cellular resources from the safety of a membrane-bound compartment. Bacteria use specialized secretion systems to translocate proteins into the host in order to subvert host processes and successfully establish infection [2]. Discovery of host-microbe interactions that take place at the protein level is fundamentally important for advancing our understanding of how bacterial pathogens evade the host’s defense machinery. Proteomic approaches hold the potential to accelerate identification of protein-protein interactions that play key roles during infection and could, therefore, provide a gateway into the design of new and improved therapeutics against bacterial pathogens.

The Gram-negative bacterium, *Legionella pneumophila*, is an opportunistic pathogen [3] responsible for Legionnaires’ disease, a life-threatening pneumonia with increased severity for individuals with a weakened immune system [4]. *L. pneumophila* is phagocytosed by alveolar macrophages and converts the phagosome into a replication-permissive vacuole by derailing phagosome maturation [5]. Survival and replication of *L. pneumophila* within the *Legionella*-containing vacuole (LCV) relies on the translocation of over 300 effector proteins into the host cytosol via a Type IVB secretion system (T4SS) encoded by the defect in organelle trafficking/intracellular multiplication (*dot/icm*) genes [3, 6–8]. Disruption of the T4SS results in failure to replicate within the macrophage [3]. Such mutants reside in phagosomes that enter the canonical endosomal maturation pathway.

Given *L. pneumophila*’s extensive arsenal of effector proteins and the functional redundancy among these effectors [9], it has been challenging to define which effectors are important for evasion of phagosome maturation. AnkX is one of the *L. pneumophila* effector proteins reported to contribute to evasion of phagolysosomal fusion [10, 11]. AnkX is a 949 amino acid protein comprising an N-terminal FIC (Filamentation induced by cAMP) domain responsible for catalyzing Rab GTPase phosphocholination, the covalent addition of a phosphocholine moiety to a serine or threonine residue; both Rab1 and Rab35 have been shown to be modified by AnkX [12]. In the absence of AnkX, the LCV accumulates lysosomal markers within 2 h post-infection, indicating that this mutant is defective in evasion of phagosome maturation [10, 11]. AnkX’s phosphocholination activity is important for preventing fusion of the LCV with lysosomes [11]. In addition to the FIC domain, AnkX harbors 13 eukaryotic-like ankyrin repeats, elements that are commonly involved in protein-protein interaction in eukaryotic cells [13]. However, information on host proteins targeted by these ankyrin repeats is not currently available.

In the present study, we aimed to further characterize the function of AnkX by identifying previously unrecognized host targets. We identified multiple interaction candidates of AnkX, and showed that PLEKHN1 associated directly with AnkX on vesicular structures. To determine the cellular pathways where PLEKHN1 might function, we capitalized on NAPPA’s ability to reveal novel interactions for this poorly characterized protein. Our results suggest that PLEKHN1 interacts with proteins involved in the inflammatory response, a host pathway commonly manipulated by microbial pathogens [14–16].

## Methods

### Strains, media and reagents

The HeLa cells were cultured at 37 °C with 5% CO_2_ in RPMI 1640 medium supplemented with 2 mM L-glutamine and 10% FBS. The COS-1 and HEK293T cell lines were cultured in DMEM medium supplemented with 2 mM L-glutamine and 10% FBS. A custom polyclonal rabbit antibody directed against AnkX (Lpg0695) was purchased from GenScript who generated the antibody by immunizing rabbits with a 14 amino acid peptide of AnkX (amino acids 32–45).

### Discovery of AnkX host targets high-density NAPPA human arrays

Five NAPPA arrays containing 10,000 purified human cDNA plasmids were printed as previously described (~2000 ORFs/slide, four C-terminal GST tagged arrays and one N-terminal Flag tag array) [17, 18]. Prior to the preparation of NAPPA protein arrays, the slides printed with plasmid DNA were blocked with Superblock solution (ThermoFisher Scientific) for 1 h at 23 °C. Then a hybridization chamber was covered on a slide in 160 μL human HeLa lysates based cell-free expression system (ThermoFisher Scientific). The in vitro transcription and translation of recombinant proteins was performed for 1.5 h at 30 °C and 0.5 h at 15 °C. After briefly washing with PBST (PBS, 0.2%Tween), the resulting NAPPA protein arrays were blocked with a PPI blocking buffer (1 × PBS, 1%Tween 20 and 1% BSA, pH 7.4) for 2 h at 4 °C. In parallel, the recombinant AnkX protein containing a C-terminal HaloTag was produced in 170 μL human cell-free expression system for 2 h at 30 °C with 100 ng/μL DNA.

To identify the host targets for AnkX, the human protein microarrays were probed with AnkX proteins for 16 h at 4 °C in conditions similar to those we have previously described [19]. Unbound protein was removed by washing three times with PPI washing buffer (PBS, 5 mM MgCl_2_, 0.5% Tween20, 1% BSA and 0.5% DTT, pH 7.4). Arrays were then incubated with 12.5 μM Alexa Fluor 660-conjugated HaloTag ligand (Promega, Madison, WI) for 2 h at 4 °C to detect human proteins interacting with AnkX. The Tecan’s PowerScanner (Männedorf) was used to obtain the fluorescent microarray images and signal intensity of each spot on microarray was quantified using the Array-Pro Analyzer software (Media Cybernetics, Bethesda, MD). Screening for interaction partners of PLEKHN1 was performed according to the experimental layout described above using PLEKHN1-HaloTag as the query protein.

### Validation of AnkX interaction proteins using bead-based pull-down assays

The bead-based pull down assay was performed as previously described (Additional file 1: Fig. S3) [18]. Briefly, 25 μL of AnkX-HaloTag was mixed with beads coated with GST-tagged host target proteins in a 96-well plate at 4 °C overnight in an Eppendorf Thermomixer® R mixer incubator at 1400 rpm. After washing three times with PPI washing buffer, protein-protein interactions were detected by incubation with Alexa Fluor 660-conjugated HaloTag ligand for 2 h at 4 °C. The complexes of AnkX and its host target proteins were eluted by boiling the beads in 20 μL 1 × SDS loading buffer containing 10% 2-mercaptoethanol. Proteins were separated by SDS-PAGE and the AnkX-HaloTag was detected using Alexa Fluor 660-conjugated HaloTag ligand, while GST-tagged bait proteins were examined by western blot using a mouse GST antibody (Cell Signaling Technology) and an HRP-labeled goat anti-mouse antibody (Jackson ImmunoResearch), respectively (Additional file 1: Fig. S2) [17, 18].

### Validation of protein-protein interactions using well nucleic acid programmable protein arrays (wNAPPA) assay

The wNAPPA assay was performed as previously described with minor modification (Additional file 1: Fig. S4) [20, 21]. Prior to the protein interaction assay, the 96-well anti-GST coated plate (GE Healthcare) was blocked with the PPI blocking buffer for 2 h at 4 °C. In parallel, AnkX and PLEKHN1 fused to a C-terminal HaloTag and their candidate target proteins fused to a C-terminal GST tag were co-expressed with human cell-free expression system in the presence of 50 ng/μL plasmid DNA. After incubating at 4 °C for 2 h, wells were washed three times with PPI washing buffer, and the protein complexes were captured by the GST antibody. Binding was detected using a chicken anti-HaloTag antibody followed by an HRP labeled anti-chicken IgG antibody (Jackson ImmunoResearch) and TMB substrate (ThermoFisher Scientific). The absorbance was measured at 450 nm.

### Construction of mammalian expression constructs

The complete list of plasmids used in this study is available in Additional file 1: Table S1. Fragments of *ankX* were cloned into 362-pCS-Cherry-DEST (Addgene plasmid #13075) using the Gateway™ cloning technology (ThermoFisher Scientific) as described before [11]. The GFP-PLEKHN1 plasmid was constructed by recombining the cDNA from pDONR plasmids (DNASU DNA plasmid repository, http://dnasu.org) into pcDNA™6.2/N-EmGFP-DEST (ThermoFisher Scientific) using the Gateway™ cloning technology. The pFN22K HaloTagCMVd1 Flexi vector (Promega) plasmid was used to clone *PLEKHN1* in order to generate an N-terminal HaloTag fusion. The pFN22K-*ankX* plasmid [11] served as template for subjected quick-change mutagenesis to generate pFN22K-*ankX*_*H229A*_ using oligonucleotides listed in Additional file 1: Table S2.

### Subcellular localization assays and confocal microscopy

HeLa or HEK293T cells were seeded onto a 12-mm cover glass (Fisherbrand™). Cells were then transiently transfected with plasmids encoding HaloTag-, GFP- or mCherry-tagged constructs (Additional file 1: Table S1) using the Lipofectamine 3000 reagent (ThermoFisher Scientific). At 10 h post-transfection, HaloTag TMR ligand (Promega, Madison, WI) was utilized for labeling of HaloTag-AnkX and AnkX^H229A^ following manufacturer’s instructions. After a PBS wash, cells were fixed by addition of 4% paraformaldehyde in PBS and incubation for 20 min at room temperature. ProLong Diamond antifade reagent (ThermoFisher Scientific) was used to mount the coversplips and images were acquired with a Zeiss 710 confocal microscope equipped with a 63× Plan-Apochromatic oil immersion objective (numerical aperture of 1.4), using the ZEN 2012 software (Carl Zeiss MicroImaging).

### Super-resolution structured illumination microscopy (SR-SIM)

SR-SIM was performed as previously described [22]. The Zeiss Elyra PSI SIM (Carl Zeiss Inc.). imaging system equipped with a 63× Plan-Apochromat oil immersion objective (numerical aperture of 1.4) was used for image acquisition. Images were generated and processed with ZEN 2011 software (Carl Zeiss Inc.) from z-stacks containing 5 phase shifts and 3 rotations per z-slice (0.110 μm interval). Alexa Fluor 488 was excited with the 488 nm laser line and emission was collected with a 495–550 nm band pass filter. Halo TMR was excited with the 561 nm laser line and emission was collected with 570–620 nm band pass filter. Affine alignment of channels was carried out using images a multicolored bead slide acquired with the same settings as the cellular sample.

### Visualization of lipid rafts at the plasma membrane

HeLa cells transiently transfected with 362-pCS-Cherry-AnkX for 18 h were placed on ice for 5 min. Subsequently, cells were incubated with ice-cold media containing 4 μg/ml Cholera Toxin Subunit B conjugated with Alexa Fluor 488 (ThermoFisher Scientific) for 5 min on ice. Cells were immediately washed with PBS and fixed with 4% paraformaldehyde at room temperature for 15 min. Cells were mounted with ProLong Gold antifade reagent (ThermoFisher Scientific) and confocal images were acquired with the Zeiss LSM 710 as described above.

### Co-immunoprecipitation experiments

HEK293T cells were transfected with plasmids encoding HaloTag-AnkX or HaloTag-AnkX_H229A_ using Lipofectamine 3000 reagent (ThermoFisher Scientific) according to the manufacturer’s instructions. Because heterologous expression of AnkX can diminish cell viability, cells were harvested at 8 h post-transfection. In parallel, HEK293T cells were transfected with plasmids encoding GFP or GFP-PLEKHN1. GFP-fusion proteins were isolated by immunoprecipitation using the GFP-Trap®_MA kit (Chromotek). Subsequently, cells were resuspended in lysis buffer provided with the kit and spun at 6000×g for 10 min to clear the cell debris. The cleared lysate was diluted with 300 μL wash buffer supplemented with 1 mM CDP-choline. A volume of 450 μL of pre-cleared lysate (corresponding to a 10cm^2^ dish of confluent cells) was incubated with 50 μL of GFP-trap magnetic agarose beads for 30 min to capture GFP or the GFP fusion proteins. The beads were first washed in the dilution buffer provided with the kit supplemented with 0.5 mM GTP, 2 mM MgCl_2_, and a protease inhibitor cocktail (Pierce Protease Inhibitor Tablets, ThermoFisher Scientific). Subsequently, these beads were incubated for 1 h with lysate of HEK293T cells expressing HaloTag-fusion proteins prepared as described above, and beads were then washed three times with the dilution buffer. Finally, the beads were boiled in 50 μL 2× Laemmli buffer to elute the proteins bound to the beads. The HaloTag-fusion lysate (input) and proteins eluted from the beads were separated on a 4–20% TGX SDS-PAGE gel (BioRad) and transferred to a PVDF membrane for immunoblot analysis. A Thermo Scientific Fast Western kit was utilized in conjunction with primary antibodies: anti-AnkX at 1:1000; anti-HaloTag (Promega, Madison, WI) at 1:1000; anti-GFP, HRP conjugated (AbCam, ab190584) at 1:10,000. The anti-rabbit secondary (ThermoFisher Scientific) at 1:5000 was used with anti-HaloTag and anti-AnkX.

### MagneHaloTag pull-down

BL21(DE3) expressing HaloTag or HaloTag-PLEKHN1 were lysed in PBS with 1 mM βME and 1 mM MgCl_2_ (supplemented with 1 mM PMSF and 0.5 mM benzamidine) using a LV1 Microfluidics and spun at 24,000×g for 35 min. Of this lysate, 500 μL were added to MagneHaloTag beads (Promega, Madison, WI) pre-equilibrated with wash buffer (PBS with 0.005% NP-40) and incubated overnight at 4 °C. The lysate was then removed and the beads were washed with wash buffer three times. 500 μL of lysate from BL21(DE3) expressing GST-AnkX prepared as indicated above was added to the HaloTag and HaloTag-PLEKHN1 coated beads and incubated for 2 h at 4 °C. The lysate was then removed and the beads were washed with wash buffer three times. The bound proteins were eluted via boiling in Laemmli buffer. The lysates and eluates were separated via SDS-PAGE and transferred to a PVDF membrane for immunoblot analysis. Fast western kit was used in conjunction with primary antibodies: anti-HaloTag (Promega) at 1:1000 and anti-GST (Thermo Scientific, MA4–004) at 1:1000.

### MagneGST pull-down

Lysate from BL21(DE3) containing GST or GST-AnkX was prepared as described above. 450 μL was added to MagneGST beads (Promega) pre-equilibrated with MagneGST binding/wash buffer and incubated for 1 h at 4 °C. The beads were then washed three times with MagneGST binding/wash buffer. 450 μL of lysate containing HaloTag-PLEKHN1, prepared as explained above, was incubated with the GST and GST-AnkX bound beads for 2 h at 4 °C. The beads were then washed three times with MagneGST binding/wash buffer and bound proteins were eluted via boiling in Laemmli buffer. Lysates and eluates were separated via SDS-PAGE and transferred to a PVDF membrane for immunoblot analysis. Immunoblot analysis was conducted with antibodies as described above.

### Subcellular fractionation

Subcellular fractionation was performed as previously described [23]. Briefly, HEK293T cells expressing HaloTag-AnkX were harvested and resuspended in PBS containing protease inhibitors. The lysate, obtained by passing the cellular suspension through a 27 gauge needle, was subjected to centrifugation at 15000×g for 10 min at 4 °C to obtain the post-nuclear supernatant (PNS). The PNS was then centrifuged at 51000 rpm in a TLA-100 for 45 min at 4 °C to obtain the cytosolic fraction. After a PBS wash to remove cytosolic contaminants, the pellet was resuspended in PBS containing 2% NP-40 to obtain the membrane fraction. The three samples, PNS, cytosolic and membrane fraction were used for further SDS-PAGE and immunoblotting analyses.

### Detergent-free separation of lipid rafts by density gradient centrifugation

Separation of lipid rafts was performed as previously described by MacDonald and Pike (2005). A total of ten 10cm^2^ tissue culture plates containing COS-1 cells transiently producing EmGFP-AnkX were washed and harvested into base buffer (20 mM Tris-HCl, pH 7.8, 250 mM sucrose). The suspension was pelleted by centrifugation for 5 min at 250×g and cells were then resuspended into 1 ml base buffer supplemented with 1 mM CaCl_2_ and 1 mM MgCl_2_ and Complete protease inhibitor cocktail (Roche) (base buffer plus). Cells were then lysed by passage through a 22 g × 3″needle 20 times and spun at 1000×g for 10 min. The postnuclear supernatant (PNS) was transferred to a separate tube and the pellet was resuspended in 1 ml base buffer plus and passaged again through a 22 g × 3″needle 20 times. Following centrifugation at 1000×g for 10 min, the PNS from the two rounds of lysis were mixed with 50% OptiPrep. The PNS mixture with a final concentration of 25% OptiPrep was placed in the bottom of a 12 ml centrifuge tube and an 8 ml discontinuous gradient of 20, 17.5, 15, 12.5, 10, 7.5, 5, and 2.5% OptiPrep in base buffer was layered on top. Samples were finally spun for 8 h at 91000×g in an SW-41 rotor in a Beckman ultracentrifuge. All the steps above were performed on ice with ice-cold buffers. Following centrifugation a total of 19 fractions were collected (0.63 ml each) and protein distribution across the fractions was assessed by Western blotting.

### Experimental design and statistical rationale

Microarray analysis was performed as we have previously described [18]. Briefly, the Array-Pro Analyzer software was used to gauge the spot shape and the presence of dust or non-specific binding in order to remove false positive signals. The normalized value was obtained by dividing the raw signal intensity of each spot by the median background-adjusted value of all features on the array. The Z-score, calculated based on this normalized value, was used to select interaction candidates as follows: (1) Z-score greater than or equal to 3; (2) Z-score ratio of query (AnkX) to the negative control (HaloTag) higher than 1.5. Interaction candidates for PLEKHN1 were selected based on the visual inspection of the luminous radiation “ring” around the microarray spot after interaction assay as described before [18, 24, 25]. This ring effect is the result of accumulation of the bait protein around the plasmid/antibody spot during IVTT and capture by the GST antibody.

Co-immunoprecipitation assays of GFP-PLEKHN1 with HaloTag-AnkX or HaloTag-AnkX_H229A_ were performed in HEK293T cells in two biological replicates. Cells ectopically expressing GFP alone were used as a control for the co-IP. Reciprocal pull-downs with GST-AnkX and HaloTag-PLEKHN1 expressed in *E. coli* were performed in two biological replicates. The GST or HaloTag alone served as the control. Co-localization of AnkX variants and PLEKHN1 was carried out in HEK293T cells.

## Results

### NAPPA-based identification of host binding partners for *L. pneumophila* effector AnkX

**Fig. 1:**
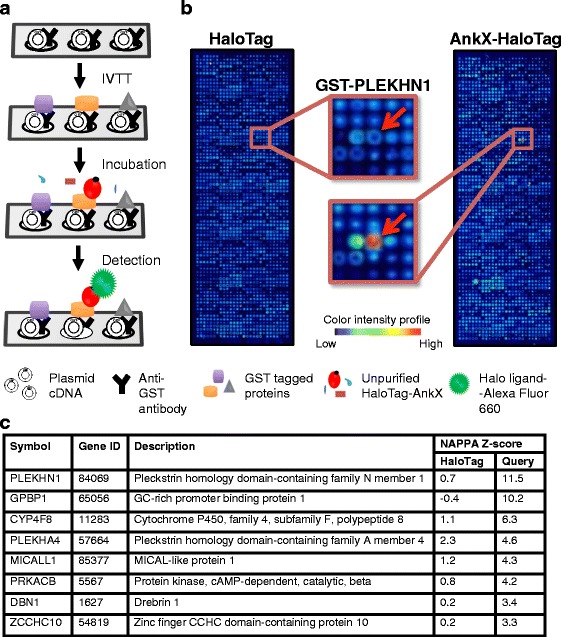
Identification of AnkX host targets by a NAPPA interaction assay. **a** Schematic illustration of the NAPPA-based protein-protein interaction screen. **b** NAPPA protein microarray after probing with HaloTag (control; left) or AnkX-HaloTag (right). Blue indicates no/low signal intensity, while red indicates maximal signal intensity. Inset shows a magnified view of the NAPPA regions containing the GST-PLEKHN1 spots (arrows) illuminated after incubation with the Alexa Fluor 660-conjugated HaloTag ligand. **c** List of AnkX host target candidates identified by NAPPA with a Z-score above 3

To identify potential interactions between AnkX and human proteins, we used a proteome array method we recently established to efficiently and reliably pinpoint human proteins bound by *L. pneumophila* effectors [18, 26]. This approach employs NAPPA displaying thousands of human proteins originating from printed expression plasmids carrying cDNAs on aminosilane-coated slides. Gene expression is accomplished via in vitro transcription/translation (IVTT) and the newly synthesized proteins are captured *in situ* by a co-spotted anti-tag antibody [27–29]. The *Legionella* query protein fused to a C-terminal HaloTag (Promega) was detected using Alexa Fluor 660-labeled HaloTag ligand, a small chloroalkane ligand that specifically and irreversibly binds HaloTag (Fig. 1a). In a previous proof-of-concept study, we demonstrated that this approach recapitulated known Rab GTPase binding profiles of the *L. pneumophila* effectors SidM and LidA, but also revealed new interaction partners for these proteins [18], highlighting NAPPA’s ability to mirror protein-protein interactions that occur at the host-microbe interface during infection. To perform the screen for AnkX candidate binding partners, human proteome arrays were incubated with AnkX-HaloTag produced by IVTT in a HeLa cell-free expression system [18, 28]. Freshly printed arrays were assessed pre-IVTT by staining the spotted DNA with PicoGreen. The average background fluorescent signal intensity of spots without human cDNA plus two standard deviations as a cut-off indicated that, comparable to our previous reports, the success rate for protein display on NAPPA was 94.1% (Additional file 1: Figure S1B) [17, 18]. Post-IVTT the array was incubated with anti-GST or anti-Flag tag antibody to detect immobilized proteins (Additional file 1: Figure S1A). Finally, the correlation of protein production between two different microarrays was 0.901, indicating high consistency of our protein array fabrication (Additional file 1: Figure S2).

AnkX interaction candidates were identified by comparing signals generated by AnkX-HaloTag to those generated by HaloTag alone. Top candidates were selected based on two criteria: a Z-score ≥ 3 and signal-to-noise ratio ≥ 1.5. Based on these criteria our top interaction candidate for AnkX was PLEKHN1. Signal intensity at the GST-PLEKHN1 position was markedly higher in the array incubated with AnkX-HaloTag than the array incubated with HaloTag alone, as shown in Fig. 1b. In addition to PLEKHN1, seven other human proteins also fulfilled the criteria mentioned above, including GPBP1, CYP4F8, PLEKHA4, MICALL1, PRKACB, DBN1, and ZCCHC10 (Fig. 1c).

### Validation of AnkX host targets by bead-based pull-down and wNAPPA

**Fig. 2:**
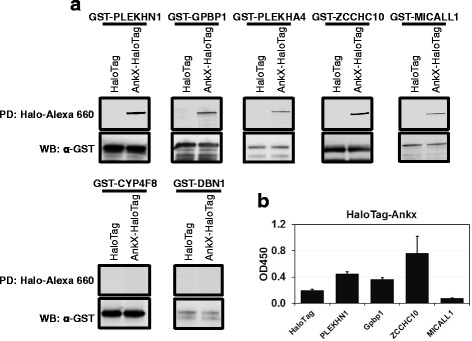
Confirmation of AnkX interaction candidates using in vitro bead-based pull-down assay. **a** Protein-protein interaction was determined using a bead-based co-precipitation assay. AnkX-HaloTag and the GST-tagged bait proteins were produced individually by IVTT. The GST-bait proteins were captured using anti-GST beads, and retention of query proteins was determined by SDS-PAGE. Top panel (pull-down, PD) shows that AnkX-HaloTag, but not HaloTag, was captured by GST-bait-coated beads. Beads coated with GST-CYP4F8 or GST-DBN1 did not capture AnkX-HaloTag. Bottom panel (western blot, WB) shows detection of the GST-bait protein by immunoblot using anti-GST antibody. **b** Protein-protein interaction was further illustrated using well-NAPPA (wNAPPA). AnkX-HaloTag and GST-bait were produced by IVTT and protein complexes were captured by well-immobilized GST antibody. Wells coated with HaloTag served as a negative control. Retention of AnkX-HaloTag was detected spectroscopically

To further probe binding of AnkX to the seven interaction candidates revealed by NAPPA, we performed an in vitro bead-based pull-down assay. Bait GST-tagged candidate proteins produced in vitro were isolated from lysate using anti-GST antibody-coated magnetic beads, and then incubated with HeLa cell lysate containing IVTT-produced query protein, AnkX-HaloTag or HaloTag alone (see schematic in Additional file 1: Figure S3). Detection of AnkX-HaloTag was performed by in-gel fluorescence using Alexa Fluor 660 conjugated HaloTag ligand and the presence of GST-tagged human proteins was validated by immunoblotting using an anti-GST antibody (Fig. 2a). The bead-based assay results provided supporting evidence for five of the eight AnkX-interaction candidates (PLEKHN1, GPBP1, PLEKHA4, MICALL1 and ZCCHC10) (Fig. 2a). The interaction of AnkX with four of these candidates was additionally validated using wNAPPA (Fig. 2b). In this approach, GST-tagged human bait proteins were immobilized in a 96-well plate and incubated with IVTT-produced AnkX-HaloTag. Our results showed that all five candidates captured AnkX-HaloTag, strongly supporting the conclusion that in vitro AnkX associates with these proteins.

### AnkX exhibits direct interaction with PLEKHN1

**Fig. 3:**
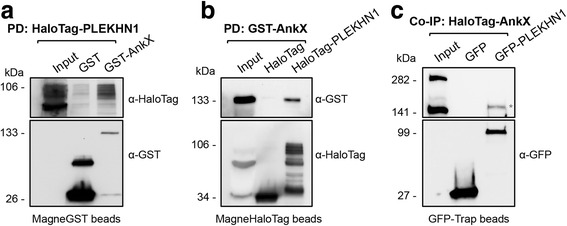
AnkX interacts with PLEKHN1 directly. **a**, **b** Reciprocal pull-downs were performed with MagneHaloTag or MagneGST beads and lysates from *E. coli* producing GST-AnkX or HaloTag-PLEKHN1. Inputs (30 μg) and eluates (43%) were analyzed via SDS-PAGE and immunoblot analysis. Anti-GST and anti-HaloTag antibodies were used to determine that GST-AnkX was captured when HaloTag-PLEKHN1, but not when HaloTag, was present on the beads. The reverse pull-down supported this result, showing that HaloTag-PLEKHN1 was precipitated with GST-AnkX, but not GST. Estimated molecular weights: HaloTag (34 kDa), HaloTag-AnkX (141 kDa), HaloTag-PLEKHN1 (106 kDa), GST (26 kDa), GST-AnkX (133 kDa), GST-PLEKHN1 (98 kDa). **c** GFP and GFP-tagged PLEKHN1 were immunoprecipitated from transiently transfected HEK293T cells using GFP-Trap™ MA beads, and incubated with lysate of HEK293T cells transiently producing HaloTag-AnkX. Inputs (~2.8%) and eluates (~60%) were separated by SDS-PAGE, and AnkX was detected by immunoblot using anti-HaloTag antibody. The bottom panel shows the GFP and GFP-PLEKHN1 present on the beads. Estimated molecular weights: HaloTag-AnkX (141 kDa), GFP (27 kDa), GFP-PLEKHN1 (94 kDa)

**Fig. 4:**
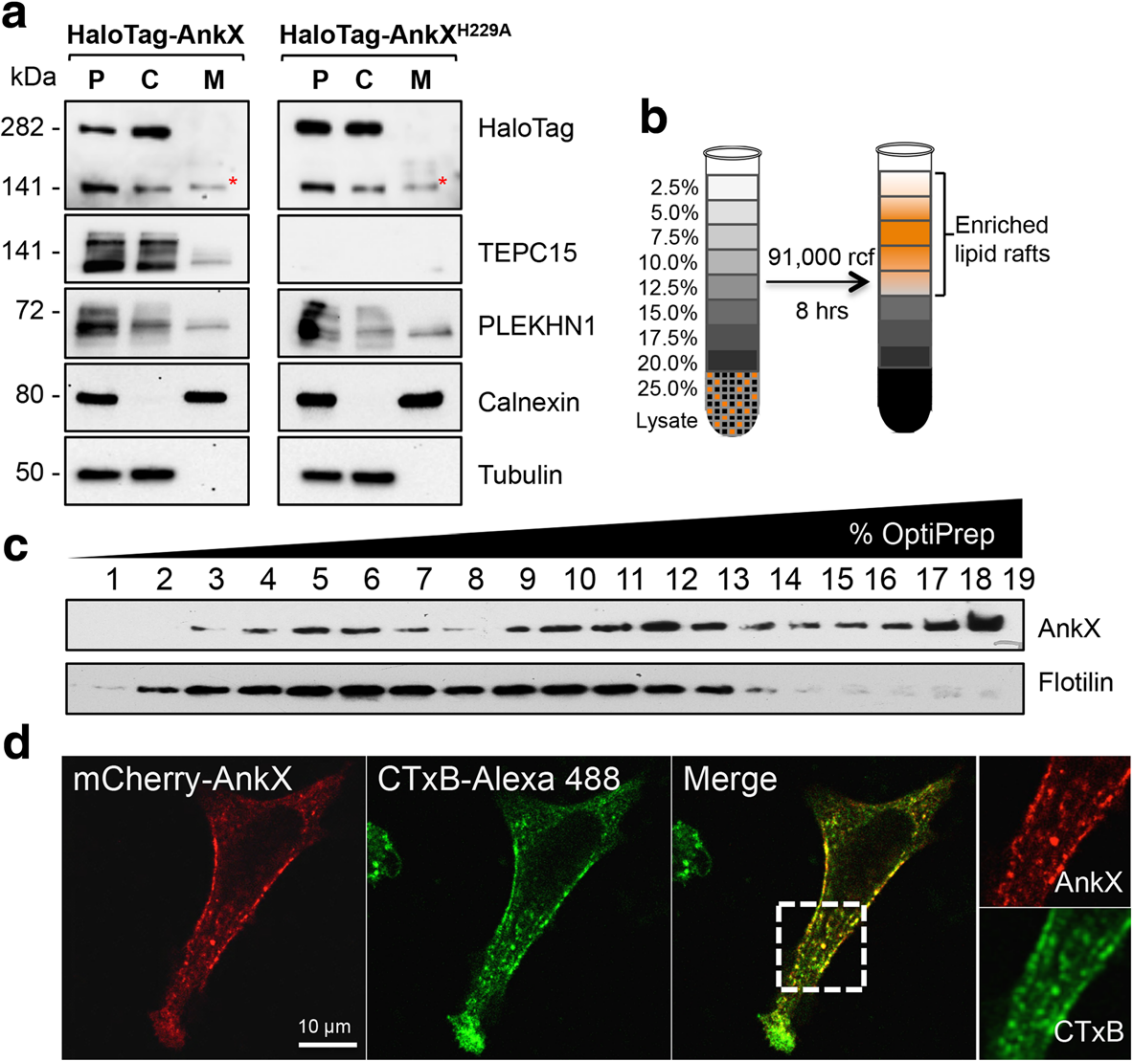
AnkX is present on membranes and co-localizes with lipid rafts. **a** Ectopically produced AnkX and endogenous PLEKHN1 localize to both the cytosolic and membrane fraction. HEK293T cells producing HaloTag-AnkX or -AnkX^H229A^ were homogenized, and the post-nuclear supernatant (P) was separated by high speed centrifugation into the membrane (M) and cytosolic (C) fraction. HaloTag-AnkX was detected by immunoblot using anti-HaloTag antibody. The TEPC15 antibody was used to detect autophosphocholination of AnkX variants. Calnexin and tubulin served as marker for the membrane and cytosolic fraction, respectively. The asterisk indicates the membrane-associated HaloTag-AnkX that is shifted upwards relative to that in the cytosolic fraction. **b** AnkX co-localizes with Cholera Toxin subunit B, a major raft marker in biological membranes. Maximum intensity projection of transiently transfected HeLa cells producing mCherry-AnkX and labeled with Cholera Toxin subunit B conjugated to Alexa Fluor 488. Cells were fixed with 4% paraformaldehyde prior to imaging by confocal microscopy. **c**, **d** GFP-AnkX is present in fractions where lipid rafts are enriched. Transiently transfected HEK293T cells producing GFP-AnkX were lysed and subjected to detergent-free isolation of lipid rafts using OptiPrep discontinuous gradient centrifugation. The presence of GFP-AnkX in the fractions collected was determined by immunoblot using an anti-AnkX antibody. A Flotilin-2 antibody was used to reveal the fractions where lipid rafts were present. All immunofluorescence images and immunoblots are representatives of at least two independent experiments with similar outcomes. Scale bar: 10 μm

Because PLEKHN1 had the highest Z-score of all candidates and was confirmed by both the bead-based pull-down and wNAPPA, we chose to focus on studying the interaction between AnkX and PLEKHN1. The assays detailed in the sections above employed only protein produced via IVTT in a HeLa cell-free system. In order to remove the possibility of a mammalian protein assisting in the interaction, reciprocal pull-downs were conducted using *E. coli* lysates containing HaloTag-PLEKHN1 or GST-AnkX. We found that GST-AnkX was successful in precipitating HaloTag-PLEKHN1, just as HaloTag-PLEKHN1 was able to form a complex with GST-AnkX (Fig. 3a and b). Therefore, our results support the conclusion that AnkX and PLEKHN1 interact directly. This conclusion was also supported by co-immunoprecipitation experiments from transiently transfected mammalian cells (Fig. 3c). GFP-PLEKHN1 or GFP (control) ectopically produced by HEK293T cells were immunoprecipitated with GFP-Trap beads and then incubated with lysate obtained from HEK293T cells ectopically producing HaloTag-AnkX. While GFP-PLEKHN1 was able to pull down HaloTag-AnkX, GFP was not (Fig. 3c). Notably, the cell lysate contained two bands for HaloTag-AnkX, one at the expected molecular weight for HaloTag-AnkX and one above 280 kDa that could be a homodimer of HaloTag-AnkX. This higher molecular weight band was not present when recombinant AnkX was produced by IVTT nor when produced in *E. coli*. Cellular fractionation assays of HEK293T cells ectopically producing HaloTag-AnkX showed that the high molecular weight band for HaloTag-AnkX (> 280 kDa) was present in the cytosolic fraction, but not in the membrane fraction (Fig. 4a). This higher molecular band was gradually reduced by increasing concentrations of SDS (Additional file 1: Figure S5).

Furthermore, HaloTag-AnkX captured by GFP-PLEKHN1 showed a shift in mass relative to the HaloTag-AnkX band in the input lane (Fig. 3c). Similarly, cellular fractionation revealed that in the membrane fraction AnkX displayed a slightly higher molecular weight than in the cytosolic fraction (Fig. 4a). Next, we asked whether this cellular distribution was dependent on AnkX’s phosphocholination activity. To address this question, we performed cellular fractionation of HEK293T cells ectopically producing HaloTag-AnkX^H229A^. We found that the catalytically inactive AnkX showed a similar distribution pattern to that obtained with the active form (Fig. 4a). Taken together, these data suggest that AnkX may assume different oligomeric states depending on whether it is membrane-associated or cytosolic.

### AnkX co-localizes with lipid rafts

In a recent study, AnkX was shown to be present on both tubular compartments and at the plasma membrane [11]. In accordance with these observations, confocal microscopy revealed that mCherry-AnkX ectopically produced in HeLa cells displayed a punctate pattern or formed distinct patches close to the cell periphery (Fig. 4d). This localization pattern resembled the distribution of lipid rafts, which form microdomains enriched in cholesterol and sphingolipids on the plasma membrane. To determine whether the mCherry-AnkX puncta/patches co-occurred with lipid rafts at the plasma membrane, we performed a co-localization assay with cholera toxin subunit B (CTxB) conjugated with Alexa Fluor 488 (Fig. 4d). CTxB attaches to the cell surface by binding to ganglioside GM1 and it is routinely used as a marker for lipid rafts [30]. Because lipid rafts are enriched at sites of endocytosis and can be internalized and then carried through the retrograde transport pathway [31], it was important to ensure that we visualized CTxB while still at the plasma membrane. Thus, HeLa cells producing mCherry-AnkX were placed on ice to halt endocytosis and then incubated with cold media containing the CTxB Alexa 488 conjugate for 5 min prior to fixing the cells. Using confocal microscopy, we observed extensive overlap between mCherry-AnkX and CTxB Alexa 488, indicating co-occurrence of mCherry-AnkX with lipid rafts (Fig. 4d). This conclusion was also supported by the biochemical isolation of lipid rafts. Detergent-free lipid rafts were isolated from COS-1 cells ectopically producing GFP-AnkX using an OptiPrep-gradient centrifugation (Fig. 4b and c). The lipid raft marker Flotillin-2 was predominantly found in the lighter fractions of the gradient and GFP-AnkX was enriched in these fractions as well. In addition, GFP-AnkX was also highly enriched in heavier fractions with low levels of lipid rafts. These results were consistent across multiple cell lines (HEK293T and HeLa) (data not shown). Together, these findings support the conclusion that AnkX co-localizes with lipid rafts at the plasma membrane and probably other intracellular membrane compartments that are not enriched in lipid rafts.

### PLEKHN1 localization and candidate interacting proteins

The biological role of PLEKHN1 (Pleckstrin homology domain-containing family N member 1) has not yet been investigated. PLEKHN1 is predicted to have two pleckstrin-homology domains in the N-terminal half, which typically recognize phosphoinositide lipids and mediate binding to specific membrane compartments. The C-terminal half of PLEKHN1 is predicted to have similarity to DNA binding domains. To gain further insight into the cellular function of PLEKHN1, we sought to determine its subcellular distribution within human cells. Super-resolution microscopy of HEK293T cells revealed that endogenous PLEKHN1 was present at multiple locations in the cell (Additional file 1: Figure S6). Based on signal intensity, endogenous PLEKHN1 was most abundant in the nucleus, where it formed speckles. In addition, PLEKHN1 was also present on small puncta throughout the cytosol and a larger vesicular structure. These puncta were likely small membrane compartments as PLEKHN1 was present in the membrane fraction of HEK293T cells ectopically producing HaloTag-AnkX (Fig. 4a).

To gain insight into the pathways that PLEKHN1 might be a part of, we aimed to identify candidate interaction partners using NAPPA (data not shown). Although none of the spots on the array with a fluorescent signal passed our internal quality criteria of a Z-score above 3, a visual inspection of the microarrays revealed several spots surrounded by a “ring”, an important feature that we have previously used to successfully identify protein-protein interaction partners, post-translational modifications, or antibody biomarkers [18, 24, 25]. We selected 10 candidates for interaction with PLEKHN1 and further analyzed those using wNAPPA (Additional file 1: Figure S7), a robust methodology for the study of protein-protein interactions [21, 32]. This approach showed that PLEKHN1 associated with BIRC4, PAK7, RNF216, NAT15, BIRC8, PSMB5, IKBKG, and TCIRG. Interestingly, several of these candidate interacting proteins are known to be involved in the inflammatory response, suggesting that AnkX may target this pathway by interacting with PLEKHN1.

### HaloTag-AnkX and endogenous PLEKHN1 co-localize on membrane compartments

**Fig. 5:**
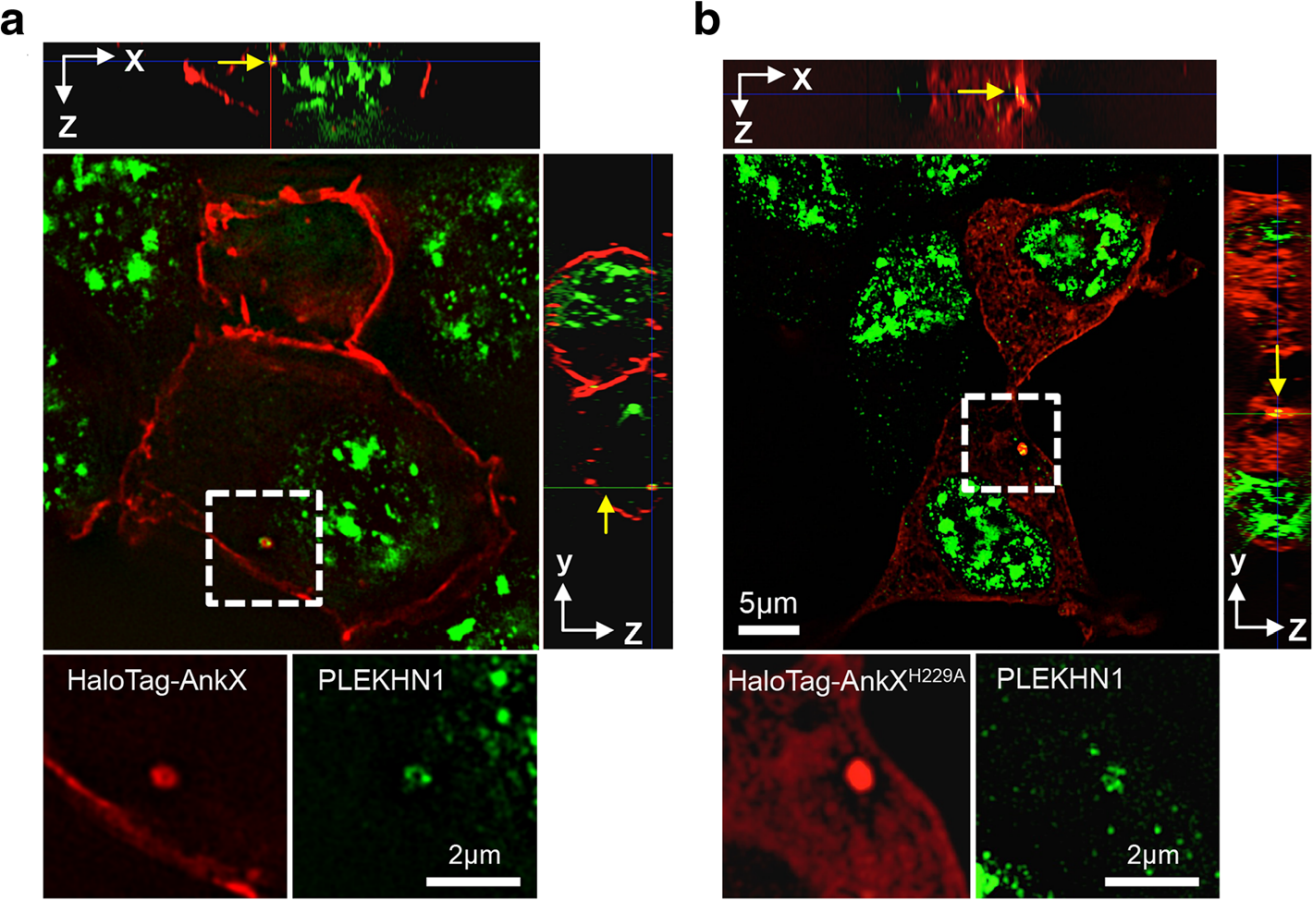
AnkX and PLEKHN1 co-localize on membrane compartments. **a**, **b** HEK293T cells were transiently transfected with plasmids encoding HaloTag-AnkX or -AnkX^H229A^. HaloTag-TMR fluorescent ligand was utilized to visualize AnkX-fusion proteins, and anti-PLEKHN1 antibody to immunostain endogenous PLEKHN1. SR-SIM images displayed co-localization between PLEKHN1 and HaloTag-AnkX on vesicular structures (marked by yellow arrows). HaloTag-AnkX^H229A^ also co-localizes with PLEKHN1 on a similar structure. Upper and side panels show orthogonal views of the HEK293T cell expressing either HaloTag-AnkX or -AnkX^H229A^ confirming the intracellular location of the vesicular structure where co-localization was observed and highlighting accumulation of HaloTag-AnkX at the plasma membrane

We next sought to determine the subcellular localization of AnkX and PLEKHN1 relative to each other. HEK293T cells ectopically producing HaloTagged AnkX or the catalytically inactive mutant, AnkX^H229A^, were stained with the HaloTag TMR fluorescent ligand and endogenous PLEKHN1 was visualized by indirect immunofluorescence microscopy. As we have observed in untransfected HEK293T cells, PLEKHN1 was abundant in nuclear speckles and was present in small puncta throughout the cell (Additional file 1: Figure S6). The subcellular distribution of active HaloTag-AnkX was different from that of the inactive mutant HaloTag-AnkX^H229A^. Active HaloTag-AnkX was present on vesicular compartments and enriched at the plasma membrane as we had previously observed (Fig. 5a). HaloTag-AnkX^H229A^ accumulated less on the plasma membrane, showing a higher signal in the cytosol and perhaps intracellular membrane compartments (Fig. 5b). Despite these differences, super-resolution microscopy revealed that both active and inactive HaloTag-AnkX co-localized with PLEKHN1 on a vesicular compartment.

### The central region of AnkX co-localizes with PLEKHN1

**Fig. 6:**
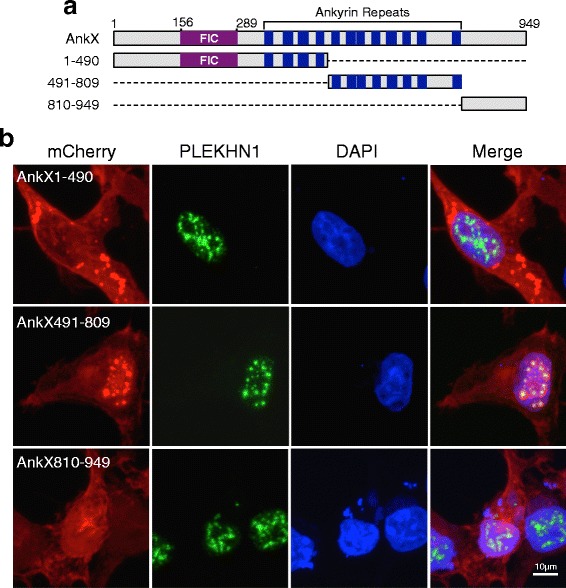
The ankyrin repeat-containing region of AnkX co-localizes with endogenous PLEKHN1. **a** Schematic representation of full-length and truncated forms of AnkX that were tagged with mCherry. **b** The central region of AnkX co-localizes with endogenous PLEKHN1. Representative confocal micrographs of HEK293T cells transiently producing mCherry-AnkX1–490, mCherry-AnkX491–809, or mCherry-AnkX810–949 and immunostained with an anti-PLEKHN1 antibody

The N-terminal FIC domain of AnkX is the only region that has been functionally characterized thus far and we next asked whether this region also mediates the interaction with PLEKHN1. We addressed this question by generating three mCherry-tagged fragments of AnkX as follows: the FIC-domain containing N-terminal region (amino acids 1–490), the ankyrin repeat-rich central region (amino acids 491–809), and the C-terminal region without any known features (amino acids 810–949) (Fig. 6a). To determine which of these fragments may co-localize with PLEKHN1 we transiently transfected HEK293T cells with the constructs encoding the three AnkX fragments above and immunostained the cells with a PLEKHN1 antibody. Confocal microscopy revealed that mCherry-AnkX1–490 did not colocalize with PLEKHN1 and nor did the mCherry-AnkX810–949 (Fig. 6b). In turn, the mCherry-AnkX491–809 fragment showed a speckled distribution in the nucleus and extensively co-localized with PLEKHN1. The central fragment also co-localized with PLEKHN1 on vesicular structures resembling those where we had observed overlap of with full-length AnkX. The N-terminal or C-terminal fragments of AnkX did not co-localize with PLEKHN1.

## Discussion

The main goal of our study was to provide additional insight into the host-pathogen interaction network that is established by the *L. pneumophila* effector protein AnkX. This protein is important during infection and its structural features, such as the presence of ankyrin repeats, suggest that it interacts with other proteins.

By applying our recently developed NAPPA screening strategy for mapping host-pathogen interactions [18], we identified eight interaction partners of AnkX (PLEKHN1, GPBP1, CYP4F8, PLEKHA4, MICALL1, PRKACB, DBN1 and ZCCHC10) (Fig. 1c). Notably, Rab GTPases were not among the top AnkX-interaction candidates identified by NAPPA. Because Rab GTPases serve as enzymatic substrates of AnkX, we speculate that their absence from our list of AnkX ligands is, at least in part, due to the transient nature of their interaction. As we have recently demonstrated, NAPPA efficiently identifies Rab GTPases bound by the *L. pneumophila* effector proteins SidM and LidA [18]. Rab binding proteins can specifically recognize either the GDP- (inactive) or the GTP-bound (active) form [33]. However, AnkX modifies its target Rab GTPases regardless of their nucleotide-bound form [34] and due to the near-physiological nature of the HeLa cell-based IVTT system it is likely that both activation states of Rab proteins are present on the array. Therefore, we presume that Rab1 and Rab35, both known substrates of AnkX, or any other Rab GTPases, did not emerge as interaction candidates because they do not form a stable complex with AnkX in the conditions provided by NAPPA. We could envision that by adapting the NAPPA approach for detection of host protein phosphocholination, Rab GTPases, and perhaps other host proteins, would be identified as AnkX substrates.

The NAPPA strategy generated multiple AnkX interaction candidates and we systematically narrowed our screen to identify high confidence candidates (Figs 1 and 2). By applying two additional approaches including a bead-based pull down assay (Fig. 2 and Additional file 1: Figure S3) and wNAPPA to validate association with AnkX, four of the eight initial candidates - PLEKHN1, GPBP1, MICALL1 and ZCCHC10 – emerged as high confidence AnkX interaction targets (Fig. 2b). Given that PLEKHN1 had the highest Z-score in NAPPA and was validated by two additional in vitro assays (Fig. 1c and Fig. 2), we considered it to be our top candidate and pursued further validation of its interaction with AnkX. Both query and bait proteins for the assays mentioned above were produced by IVTT in a HeLa cell free system, thus raising a concern that the interaction between AnkX and PLEKHN1 may not be direct. We addressed this concern by performing reciprocal pull-downs of recombinant AnkX and PLEKHN1 overproduced in *E. coli* cells and confirmed that the interaction between the two proteins is not mediated by another human protein. Therefore, we conclude that AnkX interacts with host proteins beyond its known enzymatic substrates, Rab1 and Rab35.

Within transfected cells, we discovered that HaloTag-AnkX co-localizes with endogenous PLEKHN1 on vesicular compartments (Fig. 5). The FIC-domain containing region of AnkX was not required for this interaction, instead an ankyrin-repeat rich fragment of AnkX (amino acids 491–809) co-localized with PLEKHN1 in the nucleus as well as on perinuclear vesicular structures (Fig. 6b). The subcellular localization of PLEKHN1 and full-length AnkX was rather distinct. PLEKHN1 localized in nuclear speckles, cytosolic puncta, and a vesicular compartment, whereas AnkX localized at the plasma membrane and vesicular structures. A closer look at the subcellular distribution of mCherry-AnkX revealed that it co-localized with the lipid raft marker, CTxB, at the plasma membrane (Fig. 4d). Likewise, biochemical isolation of lipid rafts from mammalian cells expressing GFP-AnkX showed that fractions enriched in lipid rafts also contained AnkX. Lipid rafts often converge at endocytic sites [35] and are enriched on recycling endosomes [36]. AnkX is known to localize on tubular compartments resembling the morphology of recycling endosomes and during host cell infection AnkX interferes with recycling endocytosis [11]. Therefore, we hypothesize that AnkX and CTxB co-localize on endocytic compartments that are either departing from the plasma membrane or recycling back to it. The finding that the catalytically inactive HaloTag-AnkX^H229A^ has a diminished presence at the plasma membrane is in line with the idea that phosphocholination of host proteins contributes to localization of AnkX at the plasma membrane. Likely there are other biochemical factors that influence AnkX’s localization at the plasma membrane. The membrane-associated AnkX had a slightly higher molecular weight than cytosolic AnkX regardless of its ability to phosphocholinate; therefore, this shift in mass was most likely not caused by autophosphocholination of AnkX. It is, thus, possible that AnkX’s association with the plasma membrane is mediated by a post-translational modification. The finding that GFP-PLEKHN1 preferentially interacted with the membrane-associated form of HaloTag-AnkX suggests that AnkX present in the membrane fraction is biochemically unique compared to cytosolic AnkX.

An important question pertains to the biological relevance of the interaction between AnkX and PLEKHN1. To gain insight into the cellular pathways where PLEKHN1 might function we used wNAPPA and identified multiple new interaction candidates. BIRC4, BIRC8, IKBKG, PSMB5 have been shown to induce gene expression driven by the transcription factor NF-κB. For instance, BIRC4 (also known as XIAP, X-linked inhibitor of apoptosis) activates NF-κB through interaction with TAB1, a regulator of TAK1 kinase activity, which functions upstream in the NF-κB pathway [37]. In addition, both BIRC4 and BIRC8 belong to the inhibitor of apoptosis (IAP) family of proteins, which are altered in many types of human cancer, and are known to regulate caspases and apoptosis, inflammatory signaling and immunity, mitogenic kinase signaling, proliferation and mitosis [38]. Furthermore, IKBKG (also known as IKKγ or NEMO, NF-κB essential modulator) plays a crucial role in activating NF-κB. IKBKG is the noncatalytic subunit of a multicomponent complex comprising two IκB kinases (IKKα and IKKβ) that phosphorylate the IκBα inhibitory subunit of NF-κB, leading to its ubiquitination and then degradation by the 26S proteasome [39]. Free NF-κB dimers translocate into the nucleus and activate expression of target genes. PSMB5 is a subunit of the catalytic core of the 26S proteasome, a ubiquitin/proteasome system that facilitates degradation of regulatory and abnormal human proteins [40]. By capturing multiple proteins that induce NF-κB activity, our NAPPA results indicate that PLEKHN1 is a component of the NF-κB signaling pathway. Whether PLEKHN1 has a positive or negative effect on activation of NF-κB remains to be established by future studies.

Remarkably, three of the four high confidence AnkX interaction candidates – PLEKHN1, GPBP1, and ZCCHC10 – localize predominantly in the nucleus, and also on vesicular structures according to data available in the Human Protein Atlas (http://www.proteinatlas.org/). The fourth AnkX interaction candidate, MICAL-L1, is an endosomal protein that interacts with Rab35 and Rab8 on recycling endosomes. This interaction profile suggests that AnkX moves from the plasma membrane through endosomes and into the nucleus. Although we did not observe nuclear localization of HaloTag-AnkX or mCherry-AnkX, it is possible that a subpool of endogenous AnkX travels into the nucleus during infection.

## Conclusions

In conclusion, our NAPPA-based methodology coupled with cellular assays and microscopy revealed previously unrecognized human interaction partners for AnkX that point to a novel role in targeting the host’s pro-inflammatory and/or anti-apoptotic pathways. Whether such interactions have an activating or an inhibitory effect on these host pathways during infection remains to be established. As an overarching conclusion, our findings highlight the multifunctional nature of *L. pneumophila* effector proteins and reassert the capacity of the NAPPA platform to provide novel insight into host-microbe interactions.

## Additional file

Additional file 1: Table S1. Microbial strains and plasmids used in this study. **Table S2.** List of oligonucleotides used for this study. **Figure S1.** Quality of NAPPA arrays in protein production. (A) Representative images of DNA PicoGreen and GST staining before and after in vitro DNA transcription and translation. (B) Distribution of fluorescent signal intensity of proteins on NAPPA microarrays. The expression rate of proteins that were displayed on NAPPA was calculated by using the signals of nonspots (buffer) plus two standard deviations. **Figure S2.** Correlation of NAPPA protein microarrays. The array contains 2206 human genes. The GST-proteins displayed on NAPPA were detected by mouse anti-GST antibody followed by HRP labeled goat anti-GST secondary antibody. **Figure S3.** Workflow of bead-based pull-down assay used in the validation of protein-protein interactions. **Figure S4.** Workflow of wNAPPA approach used for the validation of protein-protein interactions. **Figure S5.** Increasing SDS concentrations disrupt AnkX dimer formation. HEK293T cells ectopically producing HaloTag-AnkX were lysed and the post-nuclear supernatant (PNS) was collected. The PNS was incubated with increasing amounts of SDS (1.38, 1.72, 2.05, and 2.38%) in Laemmli buffer for 5 min at 80 °C. **Figure S6.** Representative SR-SIM maximum-intensity projection image displaying immunofluorescence of endogenous PLEKHN1. The subcellular distribution of PLEKHN1 is predominantly nuclear. PLEKHN1 is also found as puncta dispersed throughout the cytosol and a larger vesicular structure. Scale bar: 10 μm. **Figure S7.** PLEKHN1 interaction candidates revealed by wNAPPA. PLEKHN1 fused with a C-terminal HaloTag were co-produced with their interaction proteins using human cell-free expression system. The resulting protein complexes were captured by an anti-GST antibody-coated ELISA plate, and retention of AnkX-HaloTag was detected immunologically. These interaction proteins were selected based on the signal-to-noise ratio above 3. The HaloTag was used as a negative control. The Rab35 and LidA were employed as a positive control. (DOCX 3407 kb)

## Abbreviations

COS-1: Fibroblast-like cell line derived from African green monkey kidney
FBS: Fetal bovine serum
FIC: *Filamentation* induced by cAMP
GFP: (Emerald) green fluorescent protein
GTPase: Guanosine triphosphatase
HEK293T: Human embryonic kidney 293 cells, and contains the SV40 T-antigen
HeLa: Cell line derived from cervical cancer cells
HRP: Horseradish peroxidase
IgG: Immunoglobulin G
IVTT: In vitro transcription/translation
LCV: *Legionella*-containing vacuole
NAPPA: Nucleic acid programmable protein array
PD: Pull-down
Rab: Ras in the brain
T4SS: Type IV secretion system
