# Supplementary material for: Legionella effector AnkX interacts with host nuclear protein PLEKHN1
Source: BMC Microbiol. 2018 Jan 5;18:5. doi: 10.1186/s12866-017-1147-7 (PMC5809941; doi:10.1186/s12866-017-1147-7)
Supplement: Supplementary file 1 — Microbial strains and plasmids used in this study. Table S2. List of oligonucleotides used for this study. Figure S1. Quality of NAPPA arrays in protein production. (A) Representative images of DNA PicoGreen and GST staining before and after in vitro DNA transcription and translation. (B) Distribution of fluorescent signal intensity of proteins on NAPPA microarrays. The expression rate of proteins that were displayed on NAPPA was calculated by using the signals of nonspots (buffer) plus two standard deviations. Figure S2. Correlation of NAPPA protein microarrays. The array contains 2206 human genes. The GST-proteins displayed on NAPPA were detected by mouse anti-GST antibody followed by HRP labeled goat anti-GST secondary antibody. Figure S3. Workflow of bead-based pull-down assay used in the validation of protein-protein interactions. Figure S4. Workflow of wNAPPA approach used for the validation of protein-protein interactions. Figure S5. Increasing SDS concentrations disrupt AnkX dimer formation. HEK293T cells ectopically producing HaloTag-AnkX were lysed and the post-nuclear supernatant (PNS) was collected. The PNS was incubated with increasing amounts of SDS (1.38, 1.72, 2.05, and 2.38%) in Laemmli buffer for 5 min at 80 °C. Figure S6. Representative SR-SIM maximum-intensity projection image displaying immunofluorescence of endogenous PLEKHN1. The subcellular distribution of PLEKHN1 is predominantly nuclear. PLEKHN1 is also found as puncta dispersed throughout the cytosol and a larger vesicular structure. Scale bar: 10 μm. Figure S7. PLEKHN1 interaction candidates revealed by wNAPPA. PLEKHN1 fused with a C-terminal HaloTag were co-produced with their interaction proteins using human cell-free expression system. The resulting protein complexes were captured by an anti-GST antibody-coated ELISA plate, and retention of AnkX-HaloTag was detected immunologically. These interaction proteins were selected based on the signal-to-noise ratio above 3. The HaloTag was use [file 12866_2017_1147_MOESM1_ESM.docx]

**Additional File 1**

***Legionella* effector AnkX interacts with host nuclear protein PLEKHN1**

Xiaobo Yu^1†^, Rebecca R. Noll^2†^, Barbara P. Romero Dueñas, Samual C. Allgood^2^, Kristi Barker^3^, Jeffrey L. Caplan^2,5^, Matthias P. Machner^4^, Joshua LaBaer^3^,

Ji Qiu^3^*, M. Ramona Neunuebel^2^*

^1^ State Key Laboratory of Proteomics, Beijing Proteome Research Center, National Center for Protein Sciences-Beijing (PHOENIX Center), Beijing Institute of Radiation Medicine, Beijing, 102206, China

^2^ Department of Biological Sciences, University of Delaware, Newark, Delaware, USA, 19716

^3^Virginia G. Piper Center for Personalized Diagnostics, Biodesign Institute, Arizona State University, Tempe, Arizona, USA, 85287

^4^ Eunice Kennedy Shriver National Institute of Child Health and Human Development, National Institutes of Health, Bethesda, Maryland, USA, 20892

^5^ Delaware Biotechnology Institute, Newark, Delaware, USA, 19711

Running title: Novel human targets of the *L. pneumophila* effector AnkX

†X.Y. and R.R.N. contributed equally to this work.

To whom correspondence should be addressed:

*Dr. Ji Qiu

Arizona State University

Tempe, AZ, 85287

Tel: +1 480 9652805

E-mail: [ji.qiu@asu.edu](mailto:ji.qiu@asu.edu)

*Dr. M. Ramona Neunuebel

Department of Biological Sciences

University of Delaware

105 The Green, Newark, DE 19716

Tel: +1 302 8313450

E-mail: [neunr@udel.edu](mailto:neunr@udel.edu)

**Table S1.** Microbial strains and plasmids used in this study

| **Strain or plasmid** | **Relevant features** | **Source or reference** |
| --- | --- | --- |
| ***E. coli* strains** |  |  |
| GC5 | F− *ϕ80lacZΔM15 Δ(lacZYA-argF)U169 recA1 endA1 hsdR17(rK− mK+) phoA supE44 thi-1 gyrA96 relA1λ−tonA* | Genesee |
| KRX | [F´, *tra*D36, Δ*omp*P, *pro*A^+^B^+^, *lac*Iq, Δ(*lac*Z)M15] Δ*omp*T, *end*A1, *rec*A1, *gyr*A96 (Nal^r^), *thi*-1, *hsd*R17 (r_k_^–^, m_k_^+^), e14^–^ (McrA^–^), *rel*A1, *sup*E44, Δ(*lac*-*pro*AB), Δ(*rha*BAD)::T7 RNA polymerase | Promega |
| BL21(DE3) | F^−^*ompT hsdS_B_*(r_B_^−^ m_B_^−^) *gal dcm*(DE3) | ThermoFisher Scientific |
| **Plasmids** |  |  |
| pcDNA6.2N/EmGFP-DEST | Encodes GFP; Cam^r^, Amp^r^ | Life Technologies |
| 362 pCS-Cherry-DEST | Encodes mCherry; Cam^r^, Amp^r^ | Addgene |
| pDON221-c*PLEKHN1* | Encoding PLEKHN1; Kan^r^ | http://dnasu.org |
| pcDNA6.2N/EmGFP-DEST-c*PLEKHN1* | Encoding GFP-PLEKHN1 (human); Amp^r^ | This study |
| pFN22K HaloTag® CMV*d1* Flexi® | Encodes HaloTag; Kan^r^ | Promega |
| pFN22K HaloTag CMV*d1* Flexi-*ankX* | Encodes HaloTag-AnkX; Kan^r^ | [1] |
| 362 pCS-Cherry-DEST-*ankX*_1-140_ | Encoding mCherry-tagged AnkX fragment (aa 1-140); Amp^r^ | [1] |
| 362 pCS-Cherry-DEST-*ankX*_491-809_ | Encoding mCherry-tagged AnkX fragment (aa 491-809); Amp^r^ | [1] |
| 362 pCS-Cherry-DEST-*ankX*_810-949_ | Encoding mCherry-tagged AnkX fragment (aa 810-949); Amp^r^ | [1] |
| pFN22K HaloTag CMV*d1* Flexi-*ankX_H229A_* | Encodes HaloTag-AnkX_H229A_; Kan^r^ | This study |
| pJFT7_cHalo | Mammalian cell-free expression vector for C-terminal HaloTag fusion; Amp^r^ | [2] |
| pFN22K HaloTag CMV*d1* Flexi-*cPLEKHN1* | Encodes HaloTag-PLEKHN1; Kan^r^ | This study |

Abbreviations: Kan^r^, kanamycin resistance; Amp^r^, ampicillin resistance; Cam^r^, chloramphenicol resistance.

**Table S2.** List of oligonucleotides used for this study.

| **Name** | **Sequence 5’ to 3’ direction** |
| --- | --- |
| Fw-SgfI-PLEKHN1 | CTAAGCGATCGCCATGGGGAACAGCCACTGTG |
| Rv-PmeI-PLEKHN1 | GCCGGTTTAAACTCAGATCCACTGCACAAGCC |
| Fw-AnkX H229A | CATATTCGAATGTACGAAGTATTAGCCCCTTTTCGA |
| Rv-AnkX H229A | TCGAAAAGGGGCTAATACTTCGTACATTCGAATATG |

**
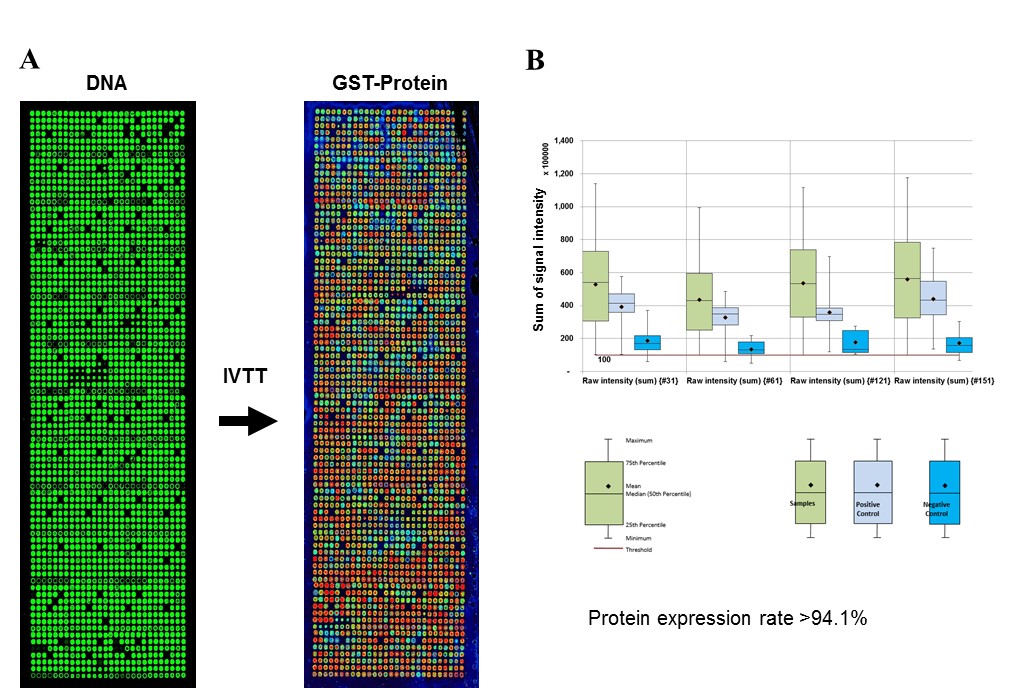
**

**Figure 3.** Quality of NAPPA arrays in protein production. (A) Representative images of DNA PicoGreen and GST staining before and after in vitro DNA transcription and translation. (B) Distribution of fluorescent signal intensity of proteins on NAPPA microarrays. The expression rate of proteins that were displayed on NAPPA was calculated by using the signals of nonspots (buffer) plus two standard deviations.

**Additional file 1.** Correlation of NAPPA protein microarrays. The array contains 2,206 human genes. The GST-proteins displayed on NAPPA were detected by mouse anti-GST antibody followed by HRP labeled goat anti-GST secondary antibody.

**
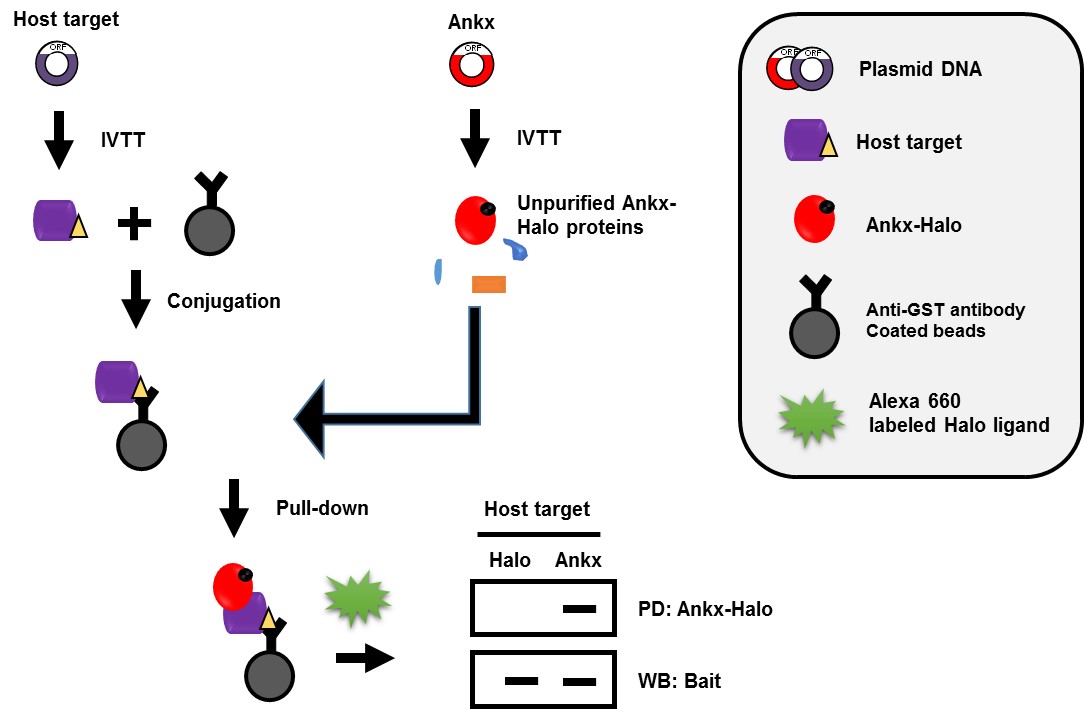
**

**Additional file 1.** Workflow of bead-based pull-down assay used in the validation of protein-protein interactions.

**
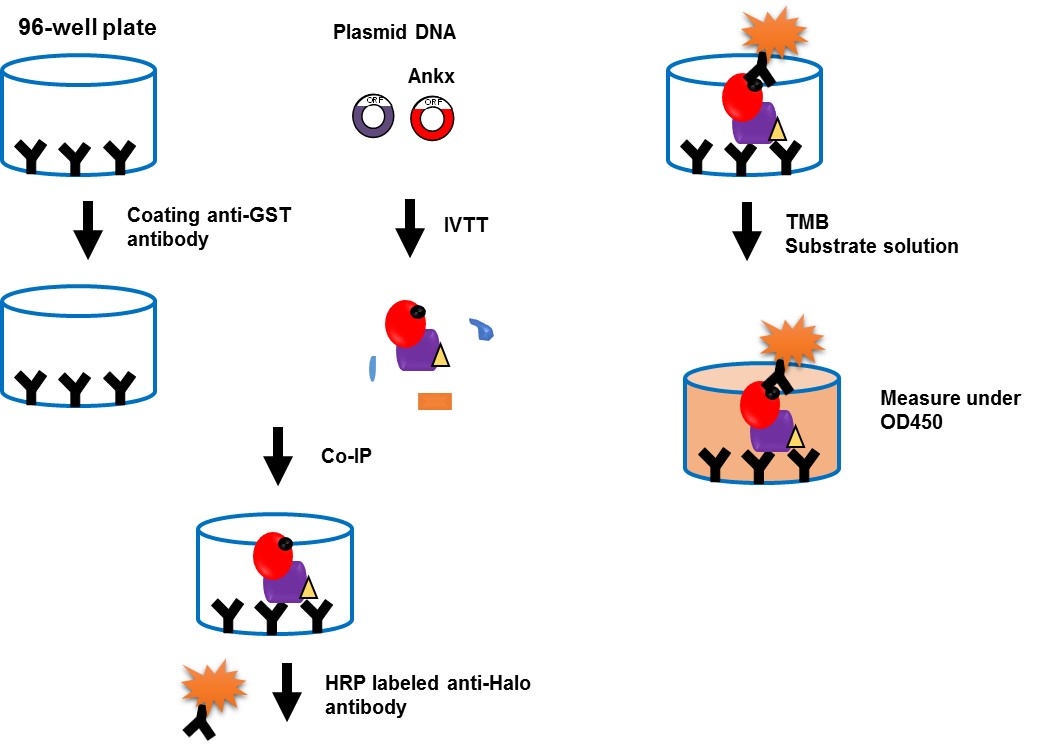
**

**Additional file 1.** Workflow of wNAPPA approach used for the validation of protein-protein interactions.

**Additional file 1.** **Increasing SDS concentrations disrupt AnkX dimer formation.** HEK293T cells ectopically producing HaloTag-AnkX were lysed and the post-nuclear supernatant (PNS) was collected. The PNS was incubated with increasing amounts of SDS (1.38, 1.72, 2.05, and 2.38%) in Laemmli buffer for 5 minutes at 80°C.

**Additional file 1.** Representative SR-SIM maximum-intensity projection image displaying immunofluorescence of endogenous PLEKHN1. The subcellular distribution of PLEKHN1 is predominantly nuclear. PLEKHN1 is also found as puncta dispersed throughout the cytosol and a larger vesicular structure. Scale bar: 10 µm

**Additional file 1.** PLEKHN1 interaction candidates revealed by wNAPPA. PLEKHN1 fused with a C-terminal HaloTag were co-produced with their interaction proteins using human cell-free expression system. The resulting protein complexes were captured by an anti-GST antibody-coated ELISA plate, and retention of AnkX-HaloTag was detected immunologically. These interaction proteins were selected based on the signal-to-noise ratio above 3. The HaloTag was used as a negative control. The Rab35 and LidA was employed as a positive control.

1. Allgood SC, Romero Dueñas BP, Noll RR, Pike C, Lein S, Neunuebel MR: **Legionella effector AnkX disrupts host cell endocytic recycling in a phosphocholination-dependent manner** *Frontiers in Cellular and Infection Microbiology* 2017, **8**.

2. Yu X, Decker KB, Barker K, Neunuebel MR, Saul J, Graves M, Westcott N, Hang H, LaBaer J, Qiu J *et al*: **Host-pathogen interaction profiling using self-assembling human protein arrays**. *J Proteome Res* 2015, **14**(4):1920-1936.
